# Super-Resolution in Plenoptic Cameras Using FPGAs

**DOI:** 10.3390/s140508669

**Published:** 2014-05-16

**Authors:** Joel Pérez, Eduardo Magdaleno, Fernando Pérez, Manuel Rodríguez, David Hernández, Jaime Corrales

**Affiliations:** 1 Department of Fundamental and Experimental Electronic, Physics and Systems, Universidad de La Laguna, Avd. Francisco Sanchez s/n, 38203 La Laguna, Spain; E-Mails: jperizq@ull.es (J.P.); mrvalido@ull.es (M.R.); dhernane@ull.es (D.H.); jaime.abraham.08@ull.edu.es (J.C.); 2 Department of Statistics, Operations Research and Computation, Universidad de La Laguna, Avd. Francisco Sanchez s/n, 38203 La Laguna, Spain; E-Mail: fdoperez@ull.es

**Keywords:** plenoptic cameras, lightfield, field programmable graphic array (FPGA), super-resolution

## Abstract

Plenoptic cameras are a new type of sensor that extend the possibilities of current commercial cameras allowing 3D refocusing or the capture of 3D depths. One of the limitations of plenoptic cameras is their limited spatial resolution. In this paper we describe a fast, specialized hardware implementation of a super-resolution algorithm for plenoptic cameras. The algorithm has been designed for field programmable graphic array (FPGA) devices using VHDL (very high speed integrated circuit (VHSIC) hardware description language). With this technology, we obtain an acceleration of several orders of magnitude using its extremely high-performance signal processing capability through parallelism and pipeline architecture. The system has been developed using generics of the VHDL language. This allows a very versatile and parameterizable system. The system user can easily modify parameters such as data width, number of microlenses of the plenoptic camera, their size and shape, and the super-resolution factor. The speed of the algorithm in FPGA has been successfully compared with the execution using a conventional computer for several image sizes and different 3D refocusing planes.

## 1. Introduction

Human beings live in a world of images, a stream that surrounds us every day so it is difficult to imagine life without them. Over the last centuries, there is evidence of the continuous evolution of devices to capture images in its design, size, and optical components. Simultaneously new and sophisticated techniques have been developed for image processing leading to a better experience for the user and to more realistic results. Precisely at the junction between photography and computers appears the Computational Photography [1] field that refers to the capture, processing, and manipulation of images that improve the capabilities of photography using computers. Computational Photography combines digital sensors, modern optics, actuators and intelligent lighting to overcome the limitations of traditional cameras. One of the drawbacks present in most of current cameras is the capture of a 2D projection of the 3D scene. To overcome this limitation and to capture a “real” image of the world, several 3D cameras have been developed. They allow viewing an object, a place or scene considering its three dimensions: height, width and depth. Using this technique, the real space with the objects and their volumes are described using three coordinate axes, two for the 2D planar spatial coordinates and one for depth. The plenoptic camera is a device that makes possible to obtain images focused at several 3D positions after the camera shot (from a captured plenoptic image or lightfield), which has brought a revolution in the photography field. A lightfield describes the light flowing along all rays in three-dimensional (3D) space. A plenoptic camera captures the 4D lightfield modifying the design of conventional cameras by inserting a microlens array between the lens of the camera and the image sensor to measure the radiance and direction of all the light rays in a scene. Conventional 2D photographs focused on determined 3D positions are obtained by 2D projections of the 4D lightfield. The fundamental ideas behind the use of plenoptic cameras can be traced back to the previous century with the works of Lippmann and Ives on integral photography [2,3]. More recently, one of the first plenoptic camera based on the principles of integral photography was proposed in Computer Vision by Adelson and Wang [4] to infer depth from a single image. In their design, the plenoptic camera consisted of a camera body with a single main lens, a microlens array in front of the camera sensor, and a relay lens to form the image on the sensor. A fundamental impulse to plenoptic cameras was given by Ng *et al.* [5] that presented a similar design, but produced in a portable hand-held device. Plenoptic cameras have left the research laboratories and some commercial models from several companies [6,7] can now be found in the market.

Unlike their conventional counterpart, plenoptic cameras capture light beams instead of dots or pixels of information. This means that when we capture a photograph, we obtain different perspectives or views of the world allowing the user to refocus the image after the shot, to obtain 3D information or to select between different views of the image.

An important drawback of plenoptic cameras is the generation of low-resolution images due to their spatio-angular lightfield sampling [8]. The angular information that is recorded reduces the spatial resolution and results in a small output image in comparison with sensor size. Methods based on super-resolution techniques that overcome this limitation in spatial resolution have recently been presented [9,10]; however, such methods are too slow to be executed in real time on an ordinary CPU. A solution for real-time processing is the use of dedicated hardware that increases the computational processing power. In recent years, field programmable graphic array (FPGA) have been used to perform general purpose computations in sensor development for telecommunications, networking, or consumer and industrial applications with a significant speedup. The low cost of the FPGA implementation and its low-consumption of energy makes this solution attractive for an implementation embedded in plenoptic cameras. In this article we describe an implementation on FPGAs of the super-resolution algorithm for plenoptic cameras presented in [10]. Currently, the same problem is being solved using other technologies based on systolic and GPU architectures [11–14].

This work is structured in five sections including this Introduction. First, we describe the theoretical background of the super-resolution algorithm. Then, Section 3 describes the design of the architecture using FPGA. Section 4 explains the experimental results and finally conclusions and future work are presented in Section 5.

## 2. Background

**Figure 1.**
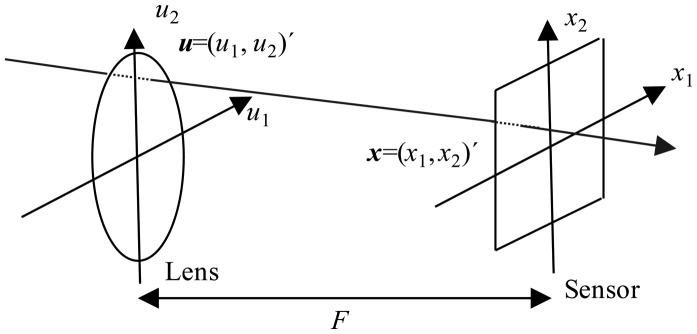
Two plane parameterization of the lightfield.

Conventional 2D photographs in a plenoptic camera are obtained theoretically using the photography transform. This transform takes a 4D lightfield as its input and generates a photograph focused on a determined plane [5]. To introduce the photography transform we parameterize the lightfield defined by all the light rays inside a camera. We will use the two-plane parameterization of this lightfield and write *L_F_*(*x,u*) as the radiance travelling from position *u* = (*u*_1_, *u*_2_)′ (apostrophe denotes transpose) on the lens plane to position *x* = (*x*_1_, *x*_2_)′ on the sensor plane. *F* is the distance between the lens and the sensor (see Figure 1 adapted from [5]).

The lightfield *L_F_* can be used to compute conventional photographs by virtually placing the sensor plane at any distance α*F* (see Figure 1). Let 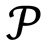
(*L_F_*) be the operator that transforms a lightfield *L_F_* at a sensor depth *F* into a photograph formed at sensor depth α*F*. The operator can be formulated as:

(1) $${\text{P}}_{\alpha }\left[L_{F}\right]\left(t\right)=\frac{1}{\alpha^{2}F^{2}}\int L_{F}\left(u\left(1-\frac{1}{\alpha }\right)+t,u\right)du$$

This equation explains how to compute a photograph 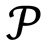
[*L_F_*] formed at a virtual sensor plane that is located at distance α*F* from the lens plane. Points in the 2D photograph depend on the spatial variable *t*. When we compute the photographs for every distance α*F* we obtain the focal stack transform of the lightfield.

Without loss of generality and to simplify the exposition we will absorb the constant term 1/*F*^2^ into *L_F_*. For rendering purposes we want the photograph taken from a constant lightfield to be a constant independent of *α* so we normalize the photography operator removing the 1/α^2^ term. Also, in order to make the discretization easier we reparametrize the focusing distance using (1 − 1/α) = *q* leading to the normalized focal stack defined in terms of the normalized photography operator as:

(2) $$P_{q}[L]\left(t\right)=\int^{}L\left(uq+t,u\right)du$$

**Figure 2.**
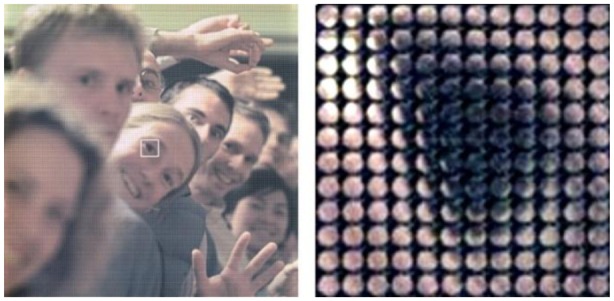
Plenoptic image and details from the white square in the plenoptic images [5].

In practice, the continuous formulation of the photography operator cannot be used since a plenoptic camera only captures discrete samples of the lightfield (Figure 2). To discretize *P*[*L*] we need to resample the lightfield and to approximate the integration process.

A simple discretization of *P_q_*[*L*] could be done by resampling the lightfield through local interpolation and replacing the integration process with sums. In this approach, the Discrete Photography Operator $P_{q}^{\text{DFS}}$ is defined as follows [10] for a lightfield *L*(*x,u*) sampled in a 4D grid = Δ*xx̃, x̃*= −*n_x_* … *n_x_*,, *u* = Δ*uũ, ũ*= −*n_u_* … *n_u_*:

(3) $$P_{q}^{\text{DFS}}[L]\left(t\right)=\sum_{\begin{array}{c} ũ=-n_{u} \end{array}}^{n_{u}}L\left(uq+t,u\right)\Delta u,\;\Delta u=\Delta u^{2}$$

with *n_x_* =(*n*_*x*1_, *n*_*x*2_) and *n_u_* =(*n*_*u*1_, *n*_*u*2_). *N_x_* = 2*n_x_* + 1 is the number of microlenses and *N_u_* = 2*n_u_* + 1 is the number of pixels behind each microlens. Variables with a tilde denote integer variables. In order to simplify the notation we will assume that *n_x_* =(*n_x_, n_x_*) and *n_u_* =(*n_u_, n_u_*). The generalization to the non-equality case is straightforward. To compute $P_{q}^{\text{DFS}}$ we need to obtain values of *L* for values that are not in the 4D grid. If we use nearest neighbour interpolation we have for *t* = Δ*tt̃*:

(4) $$P_{q}^{\text{DFS}}[L]\left(\Delta t\tilde{t}\right)=\frac{\sum_{\tilde{x}=-n_{x}}^{n_{x}}\sum_{ũ=-n_{u}}^{n_{u}}L\left(x,u\right)K_{NN}\left(\frac{x-uq-t}{\Delta t}\right)}{\sum_{\tilde{x}=-n_{x}}^{n_{x}}\sum_{ũ=-n_{u}}^{n_{u}}K_{NN}\left(\frac{x-uq-t}{\Delta t}\right)}$$

with *K_NN_* = (*x*) = 1 if *x* ≤ 1/2 and 0 elsewhere. Now, since division by Δ*t* implies that $K_{NN}\left(\frac{x-uq-t}{\Delta t}\right)=K_{NN}\left(d\tilde{x}-ũq^{\prime }-\tilde{t}\right)$ where *d* = Δ*x*/Δ*t* is the super-resolution factor, *q*′ = *q*/Δ*t* is the corrected focusing distance, the algorithm to obtain the Discrete Photography transform at points *t̃* and focused at a distance *q*′ is shown in Algorithm 1.

When the super-resolution factor *d* is an integer, round (*dx̃* − *ũ* *q*′)=*dx̃* − round (*ũq*′) and the positions for all *t̃* can be easily precomputed.



**Algorithm 1** Pseudo-code for the algorithm
**for** all *x̃* (Microlenses loop)  **for** all *ũ* (Pixels loop)   Compute *t̃* = round(*dx̃* − *ũq*′)   Update numerator(*t̃*) = numerator(*t̃*) + *L*(*x̃, ũ*)  Update denominator(*t̃*) = denominator(*t̃*) + 1 **endfor****endfor****for** all *t̃* Compute 
$P_{q}^{\text{DFS}}[L](\Delta t\tilde{t})=\text{numerator}(\tilde{t})/\text{denominator}(\tilde{t})$**endfor**


## 3. Algorithm to Hardware

The FPGA implementation of the algorithm is based on Equation (4). The Matlab-code for the super-resolution algorithm is shown in Algorithm 2.



**Algorithm 2** Matlab-code for the algorithm
%d is the super-resolution factor%qp is the 3-D focusing plane%np+1 is the center of the super-resolved output image%num is the numerator of Equation (4) and den is the denominator of Equation (4)%lf is the input lightfield and onesml is a lightfield with all its %values set to one%ret is the output super-resolved imageprecomputed_position=round(d*qp* [-nu:nu]);**for** x=-nx:nx posx=x*d+precomputed_position+np+1; %New position in the “x” microlens **for** y=-nx:nx  posy=y*d+precomputed_position+np+1;  %New position in the “y” microlens  **for** u=1:Nu   **for** v=1:Nu    num(posx(u),posy(v))=num(posx(u),posy(v))++ lf(x+nx+1,y+nx+1,u,v);    den(posx(u),posy(v))= den(posx(u),posy(v))+oneml(u,v);   **end**  **end** **end****end**ret=double(num)./double(den); %element-wise division


The Matlab-code loops each (*u, v*) pixel of each (*x, y*) microlens. Thus, the algorithm calculates, for each image data which pixel in the output image to contribute using *posx* and *posy* signals. These positions depend on pre-computed positions *posx* and *posy*, the super-resolution factor *d* and the selected slope *qp*.

In the innermost part of the algorithm is computed the contribution of each incoming data to the output image by adding its value with the existing data in the *num* matrix and taking account this contribution in the *den* matrix (which keeps track of the number of contributions for all pixels in the output image) in order to normalize the image. This last step is performed on the last for-loop for the three colors and requires the implementation of a division. We have called *Stage 2* to this last step.

The algorithm can be accelerated using parallel processing power of FPGAs instead of other classical technology platforms [15,16]. In our implementation the improvements are due to the fact that:

- Arithmetic computations are performed in pipeline and as parallel as possible.
- The algorithm is implemented fully in parallel for each color component.
- We have used a hardware divider that performs the division operation for the normalization task in a single clock cycle.

Furthermore, the algorithm implementation using FPGA offers the following additional advantages:

- Independence of the technology. The algorithm is fully implemented in VHDL. Thus it can be implemented on any FPGA from any vendor provided that meets the hardware requirements.
- The implemented system is modular and scalable because we have used VHDL generics and packages. The implemented system is easily reconfigurable by using these resources of the language. We can easily change the image size, the width of input data and intermediate calculations, the slope and the number of microlenses of the sensor.

**Figure 3.**
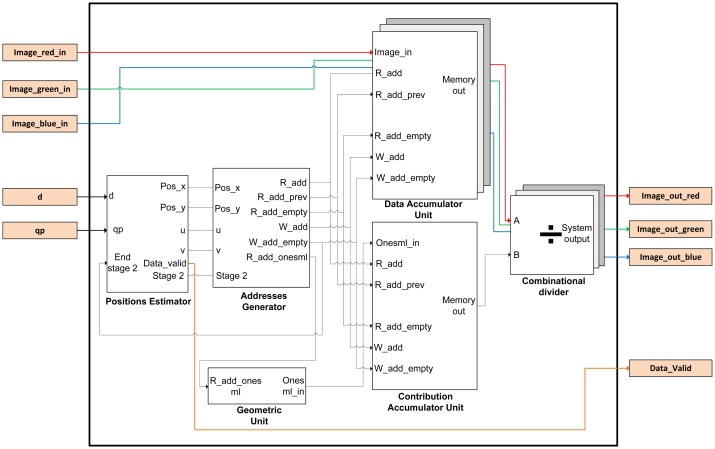
Architecture of the designed system.

The image is introduced into the FPGA by rows as a conventional image. This involves rearranging the nested loops in Algorithm 1. So, the order of the indexes has to change from *(x,y,u,v)* to *(y,v,x,u)*. In addition, the origin of the indexes of all the loops has to start at zero, because they will be implemented using hardware counters. Given these considerations, the Matlab-code has been modified as shown in Algorithm 3. Taking into account these considerations, the overall implemented architecture is depicted in Figure 3. The precomputed estimator module acts as the global controller of the system.

The functional architecture of the design has five sub-modules and a package in which is implemented the mask of microlenses used by the plenoptic sensor. This mask takes into account that microlenses vary in size and shape. The name of this component is *Geometric Unit* and it is customizable for different sizes and/or shapes of microlenses. Basically, it is a read-only memory (ROM) that stores the geometry of the microlens. The task of the *Positions Estimator* is to go over the image pixel by pixel and to compute the positions of the output image for each incoming pixel data (*posx* and *posy* signals). The *Addresses Generator* module calculates memory positions where the partial sum has to be added with the new incoming data pixel using an accumulation operation (Equation (3)). The *Data Accumulator* unit computes the *num* array in Algorithm 2 and stores the new partial data of the re-focused image in memory.



**Algorithm 3** Matlab-code for the algorithm for hardware implementation
%d is the super-resolution factor%qp is the 3-D focusing plane%np+1 is the center of the super-resolved output image%num is the numerator of Equation (4) and den is the denominator of Equation (4)%ps is the input plenoptic image and onesml is a plenoptic image with all its %values set to one%ret is the output super-resolved imageprecomputed_position=round(d*qp* [-nu:nu]);**for** y=0:Nx-1 **for** v=0:Nu-1  posy=(y-nx)*d+precomputed_position+np+1;  global_counter_y=Nu*y+v;  **for** x=0:Nx-1   posx=(x-nx)*d+precomputed_position+np+1;   **for** u=0:Nu-1    global_counter_x=Nu*x+u;    num(posx(u+1),posy(v+1))=num(posx(u+1),posy(v+1))+ ps(global_counter_x+1, global_counter_y+1);    den(posx(u+1),posy(v+1))=den(posx(u+1),posy(v+1))+onesml(u+1,v+1);   **end**  **end** **end****end** ret=double(num)./double(den);


There are three modules, one for each color component. The Contribution Accumulator unit computes the den array in Algorithm 2. It has a similar structure to the above mentioned module. Finally, the Divider module implements the normalization of each pixel in the output image by the number of contributions that apply. There is also one for each color component.

### 3.1. The Positions Estimator Module

**Figure 4.**
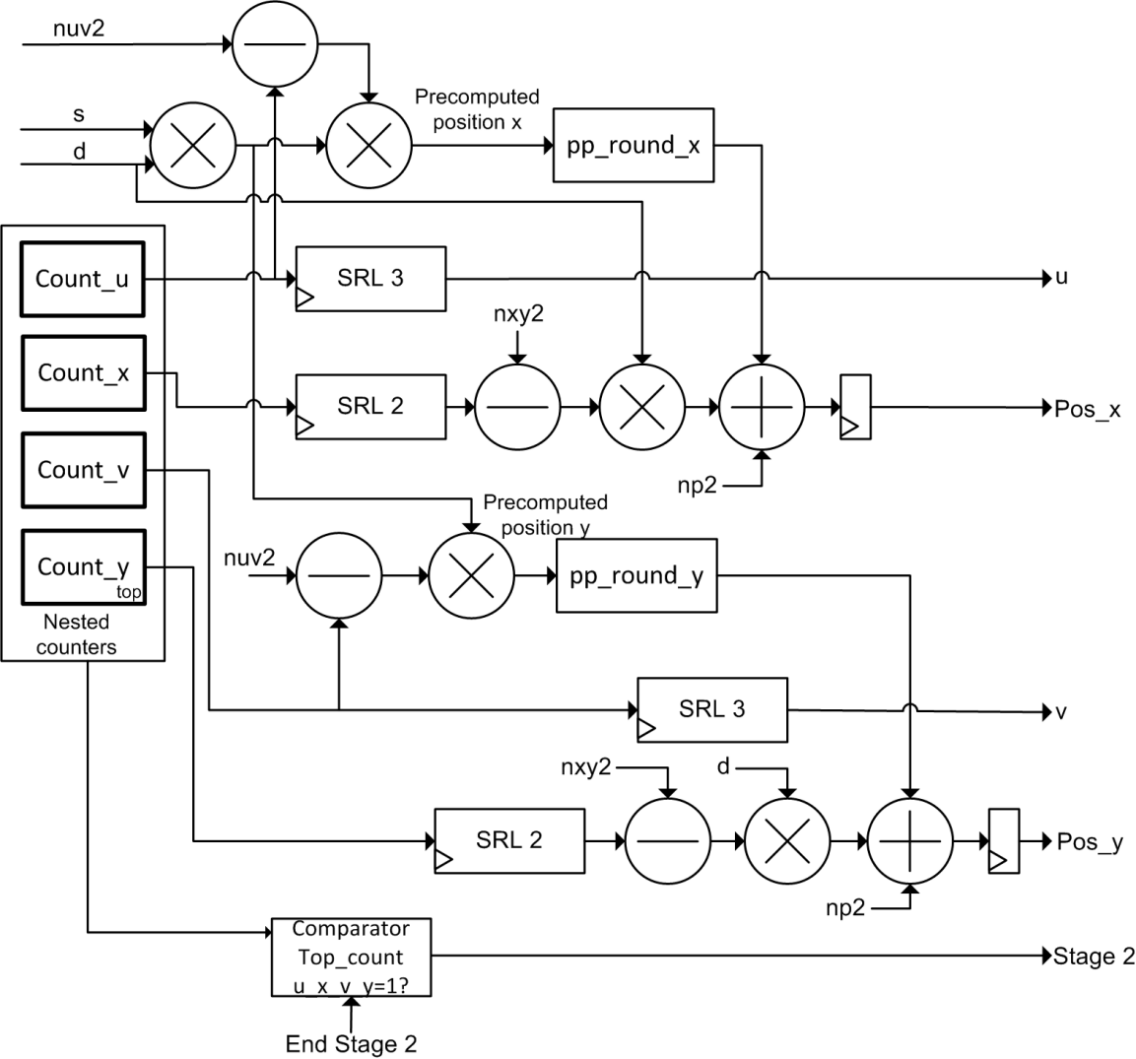
Diagram of the positions estimator module.

The implementation of this module is depicted in Figure 4. According to the algorithm, this module generates the pre-computed positions (Algorithm 1). The output positions *posx* and *posy* for each incoming data are generated based on *precomputed_positions* array, according to Algorithm 2. This module uses five clock cycles to perform the calculation of the positions.

The module contains four nested counters according to the Matlab-code of Table 3. Pixels of the lightfield are read by rows (*y,v,x,u*) using these counters as mentioned in Section 2. It also comprises a multiplier for the product of slope and super-resolution factor (*qp* and *d*), four multipliers, two stages of rounding, four subtractors and two adders to obtain *posx* and *posy* signals.

In addition, the Position Estimator module acts as the global controller of the FPGA design of the system. In this sense, it is responsible for switching the operation of the accumulation modules according to the value of the *Stage 2* signal. The evolution to Stage 2 occurs when the 4 nested counters reach the maximum value. This event indicates that all pixels of the lightfield have been accumulated. Then, the system has to realize the dump of data of the new output image (and its corresponding normalization using the divisor module). The storage modules return a finish signal when the emptying of memory is complete. Thus, it is continuously switching between the two phases of operation of the accumulators.

### 3.2. The Addresses Generator

**Figure 5.**
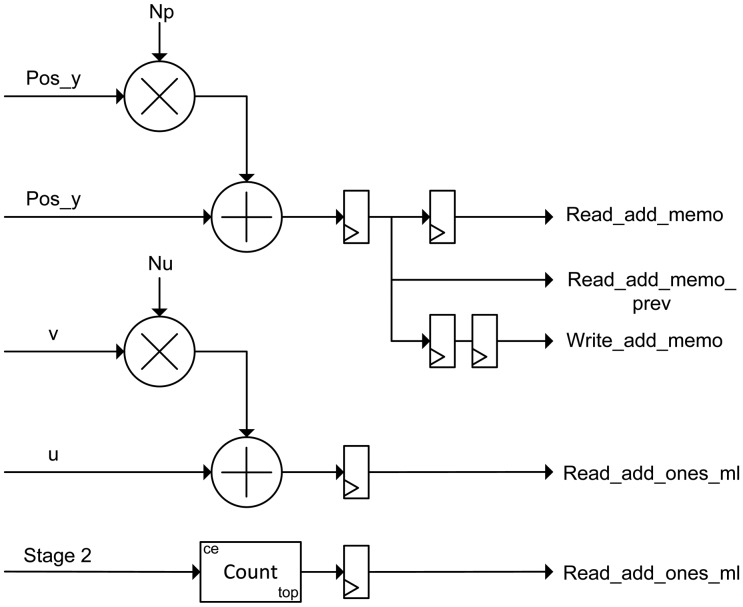
Diagram of the addresses generator.

This block is responsible for estimating the memory addresses from the pre-computed positions for reading and writing of memories, for both the accumulation stage and the memory emptying stage (Stage 2), where the memory addresses for the emptying are generated by a counter that goes from 0 to the size of the re-focused output image. The block diagram of the module is shown in Figure 5.

### 3.3. The Data Accumulator Unit

**Figure 6.**
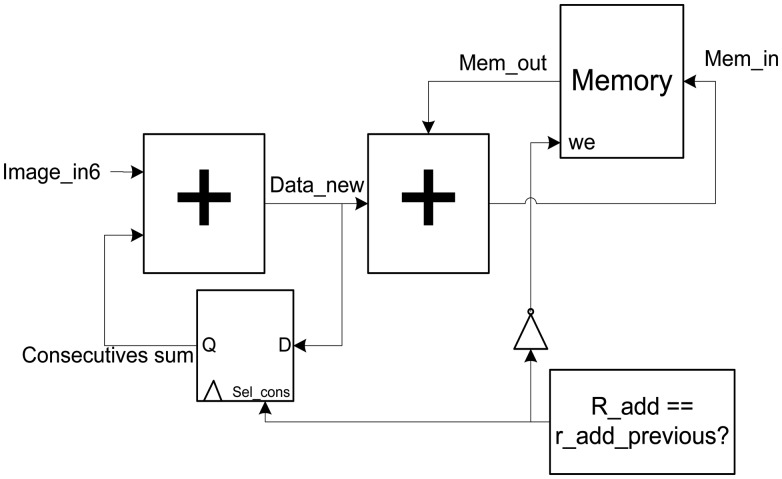
Diagram of the data accumulator.

This block is the key element of the architecture, since it makes the sum of the contributions to obtain the output image. The schematic of this module is shown in Figure 6.

The most important blocks of this module are the intermediate memory of storage and the two accumulators in series that precede it. The datum of entry (Image_in6) is delayed five clock cycles to be synchronized with the calculus of the output positions (*posx* y *posy*) and of the memory addresses that are associated with them. This supposes that in the sixth clock cycle (Included) the super-resolution algorithm begins.

#### 3.3.1. Design Considerations

An important hardware problem appears when two or more consecutives data have to be accumulated in the same output pixel. These data have associated the same values of *posx* and *posy*, and for that reason, they need to access to the same memory address. Since memory consumes one clock cycle for the reading and writing operations, the data extracted from memory for accumulation is not updated for the second consecutive data and the following arriving data.

For this reason, one accumulator is implemented to store the data which is called: “*Consecutive data*”. It is determined analyzing the actual and previous read addresses with a comparator. Both directions are obtained with a simple register. When both directions coincide, the sum of all the consecutive values is made in the first accumulator (*sum_consecutives*). In this process the line of write enable (*we* signal) is disabled through an inverter to avoid overwriting memory with wrong data. When data is no longer consecutive, the feedback register is deactivated in the first accumulator. After that, the writing operation is activated and the sum of the new accumulation (*data_new*) and the existent data which is contained in memory (*mem_out*) is performed with the second accumulator.

#### 3.3.2. Data Dumping

**Figure 7.**
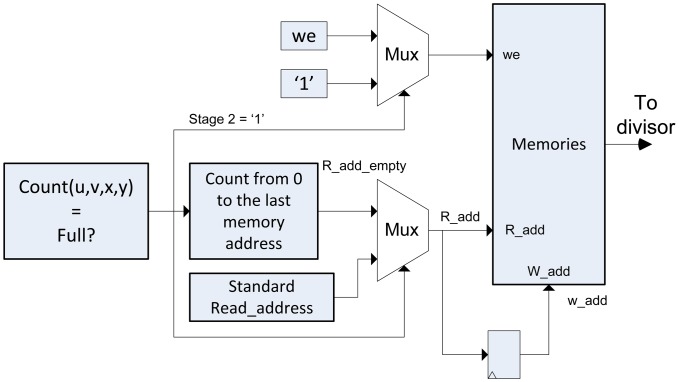
Switching between modes of accumulation and dump/erase of the memory.

After accumulating all the lightfield data, it is necessary to dump the data of the super-resolved image in a process called *Stage 2*. In this case, the reading of the memory data is performed consecutively (Consecutive addresses). In this stage the memory is erased and prepared for the execution of the algorithm with a new lightfield input. For this reason, the writing permission of the memory has always to be activated. Then, we introduce multiplexors in the entry of *we* of the memory and in the reading and writing addresses, as it is shown in Figure 7.

If the *Stage 2* signal is 0, the algorithm in Algorithm 2 is performed. The *we* signal will have a value as mentioned in the previous section, and the memory address will be provided by the Addresses Generator module. If *Stage 2* signal is 1, the address direction multiplexor switches to a standard counter, *we* signal is 1 and the data of entry to memory is always set to 0, to erase it.

### 3.4. The Contribution Accumulator Unit

The accumulator module of contributions of the output pixels has a similar architecture to the previous accumulator. The principal difference is that the size of memory is lower, as the data that has to be stored uses a lower quantity of bits.

### 3.5. Divider Module

This is a pipeline divider which makes the division using nine clock cycles. This module makes the division between A and B, of XBits and YBits respectively, with the unique restriction XBITS ≥ YBITS (Being A and B natural numbers), obtaining each nine clock cycles the quotient (*Q*) and the remainder (*R*) [17].

It is arranged as three combinational divisor modules, one for each color channel of the image that operates when the Stage 2 of the process begins. It consists in the division of each output of the memories Red-Green-Blue (RGB) by the common contribution memory (*Contribution accumulator* memory). The remainder of the divider is used to round the quotient of the division. This allows us to deliver the system output with the same data type of the input image.

### 3.6. Geometric Unit

This module is a ROM which stores a mask with the geometry of the microlenses in the lightfield. The content of this ROM is defined in one package inside the VHDL design. In this way, it is easy to modify this ROM for the size and the shape of the microlenses defined by the user.

## 4. Results and Discussion

The design for an entry image of 341 × 341 pixels (31 × 31 microlenses) is been implemented using the hardware description language VHDL and the synthesizer Xilinx Synthesis Technology (XST) in a development board Digilent Atlys with a Spartan 6 SX6SLX FPGA. The complete system is been checked using the simulation software ModelSim (Mentor Graphics Inc., Wilsonville, OR, USA) and the hardware simulation through Matlab-Simulink Xilinx System Generator (MathWorks, Natick, MA, USA). The maximum frequency is 149.591 MHz. However, the prototype currently operates at 100 MHz, a frequency which provides the Atlys FPGA board. The critical path is located inside the Position Estimation module (count_y block, 6.685 ns).

The architecture of the design allows selecting the 3D plane (slope) where we want to re-focus the image. Furthermore, it allows choosing the super-resolution factor which is applied to the original image.

**Figure 8.**
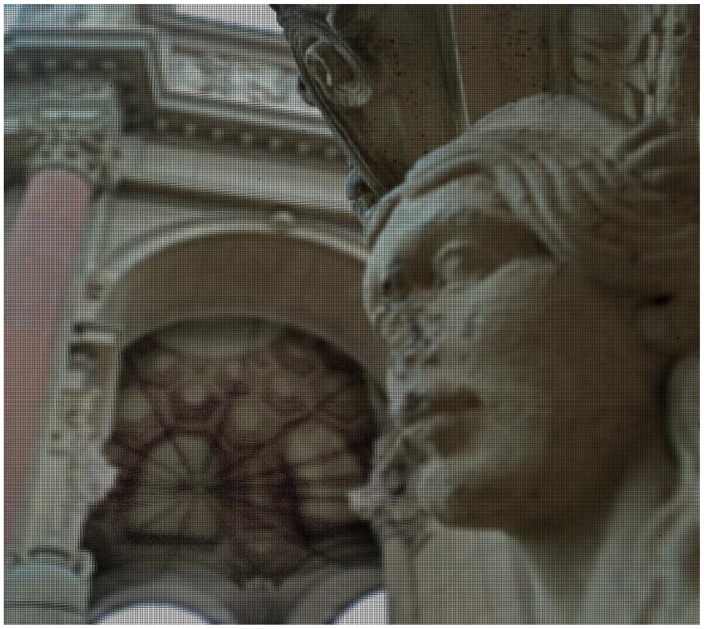
Original plenoptic image.

**Figure 9.**
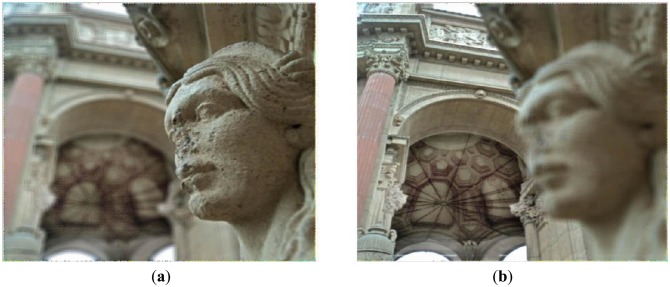
(**a**) Slope 0.4. (**b**) Slope −0.4.

Figure 8 depicts the original plenoptic image used for simulations and debugging. In Figure 9 the final results of the test image is displayed for different refocused 3D planes. We selected the lengths of the signals in the design in order to keep full precision throughout the design, avoiding overflow. In this case, the length of the incoming data is set to 8 bits. The length of the intermediate signals is such that overflow problems are avoided. The algorithm consists of accumulations of integer numbers and a final division that returns an integer quotient and an integer remainder. Thus, there is no precision loss and the resulting image is exactly the same as the integer part of the image calculated using Matlab (MathWorks, Natick, MA, USA).

**Figure 10.**
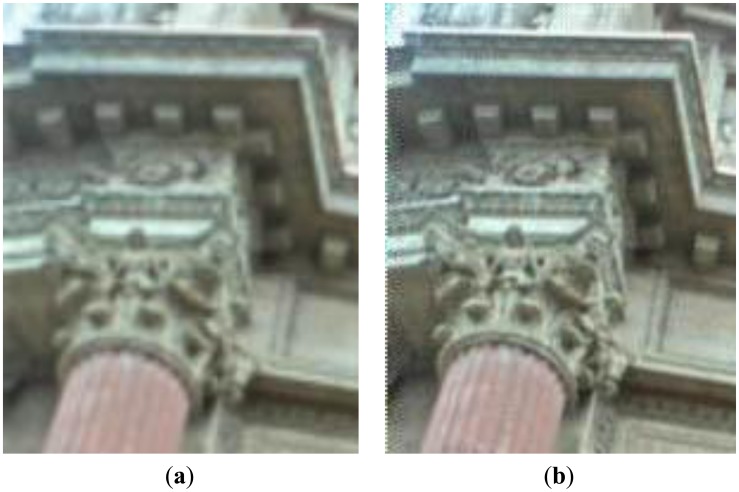
Super-resolved (*d* = 2) (**b**) and bicubic interpolation based magnification (**a**).

In Figure 10 the result of the super-resolution algorithm is displayed on the right side. This result is compared with up-sampling the refocused image without super-resolution using bicubic interpolation. The comparison shows that the super-resolution technique obtains better details in the output image.

### 4.1. Computational Time Analysis

The implemented architecture is a pipeline that permits continuous data streaming. Considering this and assuming that the throughput is the number of frames produced per unit of time, the throughput is 859.99 frames per second (116,281 cycles at 100 MHz for an entry image of 341 × 341 and 31 × 31 microlenses) and 9.76 frames per second (10,246,401 cycles at 100 MHz for an image of 3201 × 3201 pixels entry and 291 × 291 microlenses). The use of internal memory allows simultaneous accesses to the data for each color component. Furthermore, the implemented pipeline divider allows us to obtain one division for each clock cycle after the latency. The method and the technology used in this implementation is useful for mobile applications where GPUs may not be available, for instance embedded on a plenoptic camera, allowing real-time super-resolution on the camera without having to transfer the image to a host machine. Thus, the transfer from the PC to the FPGA has not been considered in the computational time analysis. Taking into account this considerations and the definition of the super-resolution algorithm, the cycles for the operation of the module are:

(5) $$N{}^{\circ}\text{cycles}=5+\left(N_{x}\;\cdot \;N_{x}\;\cdot \;N_{u}\;\cdot \;N_{u}\right)+10+\left(N_{p}\;\cdot \;N_{p}\right)=15+(N_{x}^{2}\;\cdot \;N_{u}^{2})+N_{p}^{2}$$

where:

(6) Np=2·np+1

and:

(7) $$np=\left(\frac{Nx}{2}-1\right)\;\cdot \;d+\text{round}\left(d\;\cdot \;\left|qp\right|\;\cdot \;\left(\frac{Nu}{2}-1\right)\right)$$

Independently of the plenoptic image dimensions, there is a delay of 5 cycles for calculate the values of *posx* and *posy*. The execution of the algorithm is performed in streaming, so it is necessary to take into account the size of the plenoptic image $N_{x}^{2}\;\cdot \;N_{u}^{2}$. The change of stage in the system entails an additional cycle and another nine cycles are needed for the output of the pipelined divider.

In Table 1 the time used for the super-resolution algorithm implemented in Matlab, C++, and for the entry image in the FPGA development board is shown. The size of the super-resolved output image is slightly larger than two times the number of microlenses. It is due to border effects and can be corrected if desired by cropping the final image.

For the computational time analysis in Matlab we have used a Lenovo Z580 computer (Lenovo Group Ltd., Beijing, China) with the following characteristics: Windows 7 Professional 64 bits, Intel Core i7 3612QM 2.10 GHz, 8 Gb RAM DDR3, NVIDIA GeForce GT 630 M. For the time analysis in C++ we have used a Toshiba Satellite A200-1DY (Toshiba Corporation, Tokyo, Japan) with the following characteristics: Xubuntu 12.04 32 bits, Intel Core 2 Duo T7100 1.8 GHz, 3Gb RAM DDR2-OpenCV 2.3.

Results show the improvement in the computational time of the FPGA implementation over the Matlab and C++ simulations. The time reduction factor has been 6,350 for the Images 1 and 2 (341 × 341 pixels each one), and a value of 5,415 for the Image 3 (3,201 × 3,201 pixels) in comparison with the Matlab solution and 47 and 52, respectively, in comparison with the C++ solution. The reduction factor is approximately constant since the computational complexity of the super-resolution algorithm is linear on the size of the plenoptic image. This can be verified noting that true super-resolution is only possible if the size of the output image $N_{p}^{2}$ is lower than the size of the available data $N_{x}^{2}\;\cdot \;N_{u}^{2}$ so the computational complexity in Equation (5) is bounded by $2(N_{x}^{2}\;\cdot \;N_{u}^{2})+15$. Thus, for the complete plenoptic image of 29.3 Mbytes, the FPGA processes at 276.7 Mbytes/s and is able to obtain 9 refocused planes per second. Due to the linearity in the computational complexity, increasing the operation frequency by a determined factor increases the number of refocused planes per second with the same factor.

### 4.2. Hardware Resources

Block RAMs are the critical resource for the implementation of the system in a FPGA device. Table 2 shows the memory resources used for some FPGAs. The Configuration of image column of Table 2 refers to the configuration of the memories that store the contributions of the incoming data to the pixels of the output image. Other resources such as DSP48 (arithmetic modules inside the FPGA) or slices (distributed logic elements) are always below 20% for the FPGAs under consideration.

## 5. Conclusions and Future Work

This paper presents the first FPGA implementation of a super-resolution algorithm for plenoptic sensors. The main contribution of this work is the use of FPGA technology for processing the huge amount of data from the plenoptic sensor. The algorithm execution is significantly faster than the equivalent solution on a conventional computer. This result is due to the extremely high-performance signal processing and conditioning capabilities through parallelism based on FPGA slices and arithmetic circuits and its highly flexible interconnection possibilities. Furthermore, the use of a single FPGA can meet the size requirements for a portable commercial camera.

The design of the super-resolution algorithm was developed using functional VHDL hardware description language and is technology-independent. So, the system can be implemented on any FPGA independently of its size and vendor. The design of the FPGA also makes possible to adapt the hardware to different sizes and shapes of the microlenses.

As future improvements, we are planning to implement the algorithm in bigger FPGAs, for processing plenoptic images of bigger size. The current bottleneck of the implementation is the sequential input of the plenoptic data from the charge-coupled device (CCD) to the FPGA. We are planning to use CCDs that allow parallel transfer of the image data blocks. Taking advantage of the inherent parallelism of the FPGA, and using a device large enough, the system could obtain images focused at different distances simultaneously sharing the input data. We are also considering the implementation of other algorithms based on different super-resolution interpolation kernels.

## Figures and Tables

**Table 1.** Execution time of the algorithm in Matlab.

**Parameter**	**Image 1**	**Image 2**	**Image 3**
Plenoptic input image resolution (pixels)	341 × 341	341 × 341	3201 × 3201
Number of microlenses	31 × 31	31 × 31	291 × 291
Super-resolved output image resolution (pixels)	69 × 69	69 × 69	589 × 589
Slope	0.4	−0.4	−0.4
Super-resolution (d)	2	2	2
**Total time (Matlab)**	**7.685 s**	**7.685 s**	**573.601 s**
**Total time (C ++)**	**56.9793 ms**	**56.9793 ms**	**5325.81 ms**
Time Stage 1 (FPGA)	1.162 ms	1.162 ms	102.4641 ms
Time Stage 2 (FPGA)	47.71 μs	47.71 μs	3.4693 ms
**Total time (FPGA @ 100 MHz)**	**1.210 ms**	**1.210 ms**	**105.9334 ms**

**Table 2.** FPGA internal memory resources. BRAM: Block RAM.

**FPGA device**	**Configuration of image**	**Basic internal RAM module**	**BRAM (used/available)**
XC6SLX45 Spartan 6	65 × 65 × 4	BRAM 18 Kb	28/116 (28%)
XC6VCX75T Virtex 6	65 × 65 × 4	BRAM 18 Kb	28/312 (8%)
XC6VCX75T Virtex 6	205 × 205 × 4	BRAM 36 Kb	112/156 (71%)

## References

[1] Bimber O. (2006). Computational Photography-The Next Big Step. Computer.

[2] Ives F.E. (1903). Parallax Stereogram and Process of Making Same.

[3] Lippmann G. (1908). Epreuves reversibles donnant la sensation du relief. J. Phys..

[4] Adelson E.H., Wang J.Y.A. (1992). Single lens stereo with a plenoptic camera. IEEE Trans. Pattern Anal. Mach. Intell..

[5] Ng R., Levoy M., Brédif M., Duval G., Horowitz M., Hanrahan P. (2005). Light Field Photography with a Hand-Held Plenoptic Camera.

[6] Lytro Inc.. http://www.lytro.com.

[7] Raytrix Inc.. http://www.raytrix.com.

[8] Georgiev T., Zheng K.C., Curless B., Salesin D., Nayar S., Intwala C. Spatio-angular resolution tradeoff in integral photography.

[9] Lumsdaine A., Georgiev T. (2008). Full Resolution Lightfield Rendering.

[10] Perez Nava F., Lüke J.P. Simultaneous estimation of superresolved depth and all-in-focus images from a plenoptic camera.

[11] Wimalagunarathne R., Madanayake A., Dansereau D.G., Bruton L.T. A systolic-array architecture for first-order 4-D IIR frequency-planar digital filters.

[12] Lumsdaine A., Chunev G., Georgiev T. Plenoptic rendering with interactive performance using GPUs.

[13] Hahne C., Aggoun A. Embedded FIR Filter Design for Real-Time Refocusing Using a Standard Plenoptic Video Camera.

[14] Lüke J.P., Pérez Nava F., Marichal-Hernández J.G., Rodríguez-Ramos J.M., Rosa F. (2010). Near Real-Time Estimation of Super-Resolved Depth and All-In-Focus Images from a Plenoptic Camera Using Graphics Processing Units. Int. J. Digit. Multimed. Broadcast..

[15] Magdaleno E., Rodríguez M., Rodríguez-Ramos J.M. (2010). An Efficient Pipeline Wavefront Phase Recovery for the CAFADIS Camera for Extremely Large Telescopes. Sensors.

[16] Magdaleno E., Lüke J.P., Rodríguez M., Rodríguez-Ramos J.M. (2010). Design of Belief Propagation Based on FPGA for the Multistereo CAFADIS Camera. Sensors.

[17] Sutter G., Bioul G., Deschamps J.P., Boemo E. (2004). Power Aware Dividers in FPGA. Lect. Notes Comput. Sci..

